# Molecular mechanisms of tissue inhibitor of metalloproteinase 2 in the tumor microenvironment

**DOI:** 10.1186/2052-8426-2-17

**Published:** 2014-06-03

**Authors:** Taylor C Remillard, Gennady Bratslavsky, Sandra Jensen-Taubman, William G Stetler-Stevenson, Dimitra Bourboulia

**Affiliations:** Department of Urology, SUNY Upstate Medical University, 750 East Adams Street, Syracuse, NY 13210 USA; Radiation Oncology Branch, Center for Cancer Research, National Cancer Institute, National Institutes of Health, Advanced Technology Center, 8717 Grovemont Circle, Bethesda, MD 20892-4605 USA; Department of Urology and Department of Biochemistry & Molecular Biology, SUNY Upstate Medical University, 750 East Adams Street, Syracuse, NY 13210 USA

**Keywords:** Tissue inhibitor of metalloproteinase-2 (TIMP-2), Matrix metalloproteinase (MMPs), Tumor microenvironment (TME), Angiogenesis

## Abstract

There has been a recent paradigm shift in the way we target cancer, drawing a greater focus on the role of the tumor microenvironment (TME) in cancer development, progression and metastasis. Within the TME, there is a crosstalk in signaling and communication between the malignant cells and the surrounding extracellular matrix. Matrix metalloproteinases (MMPs) are zinc-dependent endoproteases that have the ability to degrade the matrix surrounding a tumor and mediate tumor growth, angiogenesis and metastatic disease. Their endogenous inhibitors, the Tissue Inhibitors of Metalloproteinases (TIMPs), primarily function to prevent degradation of the ECM via inhibition of MMPs. However, recent studies demonstrate that TIMP family members also possess MMP-independent functions. One TIMP member in particular, TIMP-2, has many distinct properties and functions, that occur independent of MMP inhibition, including the inhibition of tumor growth and reduction of angiogenesis through decreased endothelial cell proliferation and migration. The MMP-independent molecular mechanisms and signaling pathways elicited by TIMP-2 in the TME are described in this review.

## Review

### Introduction

Historically, much of cancer research has focused on the proliferation of malignant cells and how oncogenes become activated while tumor suppressor genes become inactivated [1]. More recently, there has been a change in the ways we investigate tumors and neoplastic growth that has lead to an expansion of research interests to include a broader focus, not only on the tumor cells alone, but also on how the tumor microenvironment (TME) affects cancer initiation and progression. The TME provides great promise for the identification of new therapeutic targets and novel cancer therapies that can only be fully exploited by understanding the molecular mechanisms involved in TME regulation of tumorigenesis and the development of metastatic disease.

### MMPs and ECM degradation

The TME is composed of cellular and non-cellular components. The extracellular matrix (ECM) is the non-cellular, structural element of the TME that plays a central role in tumor cell communication. There are two types of ECM: a continuous layer of matrix that constitutes the basement membrane (BM) and the stroma or interstitial matrix. BM, a key structure of the ECM, is a subepithelial/endothelial layer of fibrous, cross-linked proteins (such as Type IV collagen), glycoproteins and associated proteoglycans. The BM provides not only physical support but also metabolic support to the cellular components (i.e. oxygenation and waste removal). The BM separates the epithelial compartment from the surrounding stromal tissue. The BM does not contain pores large enough to allow cellular transmigration. Thus, extensive proteolytic disruption and/or remodeling of the basement membrane remain the hallmark of the metastatic potential in malignant cancers. The ECM in the connective tissue (the interstitial matrix) consists of fibers (Type I collagen, reticular, and elastic fibers), ground substances (unstructured amorphous material that surrounds cells and fibers) primarily composed of glycosaminoglycans, glycoproteins and proteoglycans, as well as intersitial fluid. Within the interstitial matrix lay stromal cells including fibroblasts, pericytes and immune cells that constitute part of the cellular components of the TME. Important signaling events and communication between tumor cells and a variety of cellular components of the TME strongly influence tumor progression [2, 3].

The ECM also contains secreted proteins, produced by the tumor cells and/or activated stromal cells, which elicit signaling pathways and promote tumor development [2, 3]. Upon ECM breakdown, ECM-bound peptide growth factors, such as Insulin-like Growth Factor (IGF), vascular endothelial growth factor (VEGF) and transforming growth factor-β (TGF-β) are released [4–6]. Collagens type IV and XVIII derived fragments in the ECM such as arrestin, canstatin, tumstatin, restin, and vastatin can either have pro- or anti-angiogenic functions [7]. It is, therefore, apparent that the ECM plays major, determining role on whether cancer will develop and progress or become dormant. With respect to tumor growth, the ECM provides a supporting role, and communicates with the tumor through signaling between growth factor, cytokine and cellular adhesion receptors, making the understanding of its influence on the tumor behavior not only complex but also of great importance.

The Matrix Metalloproteinase (MMP) family members are a group of metzincin metalloproteases, along with a disintegrin and metalloproteinases (ADAMs) and ADAM proteases with thrombospondin motifs (ADAMTSs), that function primarily extracellularly, and are critical for the maintenance and remodeling of tissues, largely by degrading the ECM. MMPs are zinc-dependent endopeptidases that cleave peptide bonds on the nonterminal ends of their numerous target proteins [8, 9]. The MMPs are categorized based on whether they are secreted or remain bound to the cell membrane. Additionally, they can be organized by their structure and substrate specificity: collagen, gelatinases, membrane type, stromelysins and matrilysins (Table 1). MMPs are produced and secreted without biological activity as zymogens. MMPs contain a cysteine-sulfhydryl group in their propeptide domain that associates with a bound zinc ion in the proteins catalytic domain. This association maintains the MMPs’ zymogen form and keeps them inactive. The disruption of the interaction between the zinc ion and the cysteine residue, known as the ‘cysteine switch’, which is usually initiated by a serine protease, leads to the cleavage of a pro-peptide and the activation of the MMPs [10].

**Table 1:** **Matrix metalloproteinases (MMPs) categories**

Secreted MMPs		Membrane bound MMPs		
		Type I transmembrane	GPI- anchored	Type II transmembrane
MMP-1	MMP-2	MMP-14	MMP-17	MMP-23A
MMP-3	MMP-7	MMP-15	MMP-25	MMP-23B
MMP-8	MMP-9	MMP-16		
MMP-10	MMP-11	MMP-24		
MMP-12	MMP-13			
MMP-19	MMP-20			
MMP-21	MMP-27			
MMP-28				
**Structure and substrate specificity**				
**Collagenases**	**Gelatinases**	**Stromelysins**	**Matrilysins**	**Membrane-Type**
MMP-1	MMP-2	MMP-3	MMP-7	MMP-14
MMP-8	MMP-9	MMP-10	MMP-26	MMP-15
MMP-13		MMP-11		MMP-17
MMP-18				MMP-24
				MMP-25

MMPs have long been the focus of investigation for potential use as disease biomarkers [11]. Levels of secreted and active forms of MMPs are usually increased during cancer progression, and have been associated with the development of tumor metastasis [2]. Thus, synthetic MMP inhibitors have been evaluated as therapeutics in clinical trials for cancer treatment. Surprisingly, they proved to be largely ineffective [12]. This outcome may be mainly due to the large number of MMP family members, where the benefits of inhibiting a few are compensated by the activity of other MMPs. Furthermore, recent studies have reported that MMPs can both promote and inhibit tumor growth and progression [13].

### TIMPs and their interaction with the TME

Tissue inhibitors of matrix metalloproteinases (TIMPs) are a protein family that functions as natural MMP inhibitors, along with inhibiting the activity of ADAMs and ADAMTs families of metalloproteinases, albeit with distinct affinities [14]. Whereas three of the four TIMPs (TIMP-1, -3, and -4) are nested within the *synapsin* gene family [15], TIMP-2 contains a nested gene, *differential display clone 8* (DDC8), in its first intron [16]. There are some similarities among the TIMP family members. They each share 40% of their basic structure that their amino and carboxyl ends each contain six cysteine residues, which form 3 conserved disulfide bonds on both the N-terminal and C-terminal ends of the protein [17, 18]. The various functions of TIMPs have been described [19–21]. Recent evidence has shown that TIMPs control important cellular processes including proliferation, apoptosis and angiogenesis by mechanisms independent of their MMP inhibitory activity [14].

As its name implies, TIMPs inhibit MMP activation by direct interaction. TIMP inhibition of MMP occurs primarily through their N-domains [22]. However, a unique feature of TIMP-2 is its ability to activate MMP-2. The activation of MMP-2 occurs via a distinctive mechanism involving MT1-MMP (MMP-14), a membrane bound MMP, and TIMP-2 [23]. The N-terminus of TIMP-2 binds and inhibits MT1-MMP, whereas the C-terminus can bind to secreted pro-MMP-2 (zymogen, inactive form). Upon binding of TIMP-2 to MT1-MMP, a neighboring MMP-14 that is not inhibited (free of TIMP-2) can cleave and activate a bound pro-MMP-2. It has also been shown that this activation is accelerated if the C-terminus of MMP-2 is bound to alphav beta3 (α_v_β_3_) integrin receptor on the cell surface that is proximally located to the MT1-MMP [24]. TIMPs (and MMPs) play an active role in tumor cell adhesion [25]. Members of the TIMP family have been shown to engage with the cell surface by binding to cellular receptors; TIMP-1 interacts with CD63, a member of the tetraspanin family that interact with cell adhesion molecules, such as β_1_ integrin. This interaction results in inhibition of caspase-mediated apoptosis in mammary epithelial cells. Moreover, TIMP-2 inhibits angiogenesis by interacting with alpha3 beta1 (α_3_β_1_) integrin on endothelial cells; this occurs in an MMP independent fashion. TIMP-2 has also been shown to interact, via the C-terminal loop 6 domain, with the insulin-like growth factor-I receptor (IGF-I-R). TIMP-3 also binds to VEGF receptor 2 (VEGFR2), acting as a Vascular Endothelial Growth Factor A (VEGF-A) antagonist and blocking endothelial cell proliferation. In conclusion, TIMPs role in tumor cell growth involves a combination of mechanisms including inhibition of MMP-mediated ECM degradation and interactions with the cellular part of the TME [25].

### TIMP-2 MMP-independent functions

There are many TIMP-2 functions that take place independent of its MMP inhibitory activity [26]. In general, MMP-independent TIMP functions, including those of TIMP-2, have only recently been established and there is contradictory evidence depending on the tumor or repair mechanisms under study. This only reinforces the need for more studies on the mechanisms of TIMP MMP-independent functions in order to understand the complexities of these MMP-independent activities. There is a collection of research studies focusing on the TIMP-2 effects either on tumor cells, and how they might influence the TME, or on endothelial cells and tumor endothelium. See Table 2 for a summary of the TIMP-2 MMP-independent functions.

**Table 2:** **MMP-independent functions of TIMP-2**

Affected protein	Effect on function	Mechanism	Reference
VEGF-A	Reduced human microvascular endothelial cell (hMVECs) growth. Reduced human umbilical vein endothelial cell migration, proliferation, and tubular formation	TIMP-2 N-terminus binds to α_3_β_1_ integrin receptor. TIMP-2 peptide 9 inhibits VEGF-A, resulting in increased cAMP/PKA levels and the induction of p27^Kip1^	[27–29]
VEGFR2 and FGFR1	Decreased endothelial cell proliferation and angiogenesis	TIMP-2 binds to the α_3_β_1_ integrin receptor; SHP-1 inactivates/dephosphorylates VEGFR2 and FGFR1	[20, 27, 30]
VEGF-A	Decreased vascular permeability	Inhibition of VEGF-A leads to increased cAMP levels. Increasing VE-cadherin association with the actin cytoskeleton, increasing cell-cell contacts	[31]
Decreased ERK and AKT phosphorylation	Inhibition of endothelial cell proliferation and angiogenesis	TIMP-2 binding and inhibition of IGF-R1 via Loop 6, C-terminus	[32]
RECK and p27^Kip1^	Suppression of endothelial cell migration	Transcriptional regulation	[33, 34]
MDSCs	Reduction of angiogenesis and A549 tumor growth	TIMP-2 inhibition of the recruitment of MDSCs	[35]
EGFR	Decreased EGFR activation and growth suppression on A549 cells	Binding of TIMP-2 to increase cytosolic cAMP levels, preventing SHP-1 from dissociating EGFR, leading to hypophosphorylation and inactivation of EGFR	[36]
FAK and AKT, Decreased FAK and AKT phosphorylation	A549 cell migration and invasion. In A549 xenograft tissues by immunohistochemistry	Not determined	[37]
EFEMP1, Fibronectin, E-cadherin, IGF-R1, SEMA-3A, ANGPT1	A549 lung cancer xenografts Inhibition of tumor growth *in vivo*	Transcriptional regulation	[38]
ABCB1, ABCG2, AKR1C1	Decreased expression in side population in A549 cells, increased chemosensitivity	Transcriptional regulation	[39, 30]

#### Effects on endothelial cell function and angiogenesis

TIMP-2 can inhibit endothelial cell proliferation, independent of MMP inhibition, through binding to endothelial cell receptors, eliciting a series of signaling events [20]. We have shown that TIMP-2 inhibits endothelial cell growth mediated by angiogenic factors, including VEGF-A and Fibroblast Growth Factor 2 (FGF-2), by binding to the alpha3 beta1 integrin receptor (α_3_β_1_) on endothelial cells [27]. This leads to a decrease in the amount of protein tyrosine phosphatase (PTP), SHP-1, that associates with the β_1_ integrin subunit coupled with the increase in the dissociation of HSP60 from this same subunit. This result leads to a signaling cascade of events mediated by the inactivation of receptor tyrosine kinases via SHP-1. TIMP-2 is able to reduce this α_3_β_1_ activation by promoting the dissociation between the integrin and HSP60 [27]. TIMP-2 increases SHP-1 activity associated with FGFR1 (Fibroblast Growth Factor Receptor 1) and VEGFR2 (Vascular Endothelial Growth Factor Receptor 2), upon growth factor stimulation, leading to the belief that TIMP-2 induces a switch of SHP-1 binding from α_3_β_1_ to FGFR1 or VEGFR2, as well as an increase in SHP-1 activity. Additional studies showed that the TIMP-2 N-terminus is involved in the antiangiogenic effects and inhibited VEGF-A mediated endothelial cell growth stimulation. This N-terminus domain is solvent exposed, flexible and is unique to TIMP-2, compared to the other TIMP family members [27, 28]. More recently, the N-terminus TIMP-2 derived 18-mer peptide, peptide 9, was found to inhibit human umbilical vein endothelial cell migration, proliferation, and tubular formation via inhibition of VEGF-A-stimulated cell proliferation, leading to the induction of p27^Kip1^ from increased cAMP/protein kinase A (PKA) levels [29]. In another study, it was also shown that, in addition to its antiangiogenic effects, TIMP-2 mediated inhibition of vascular permeability via a α_3_β_1_-SHP-1-cAMP/PKA dependent mechanism involves vascular E-cadherin (VE-cadherin) [29, 31]. More specifically, TIMP-2 treatment increased VE-cadherin distribution in cell-cell contacts through increased association with the actin cytoskeleton.

TIMP-2 was also confirmed to inhibit endothelial cell proliferation and angiogenesis through its C-terminus, Loop 6 domain [32]. TIMP-2 Loop 6 inhibits downstream signaling of Insulin-like Growth Factor Receptor 1 (IGF-R1), specifically AKT and Erk phosphorylation, yet does not alter the MT1-MMP expression levels [32]. It was also shown that Loop 6 binds to IGF-R1 and initiates the downstream inhibitory signaling in endothelial cells.

Furthermore, TIMP-2 suppresses endothelial cell migration by inducing the reversion-inducing-cysteine-rich protein with Kazal motif (RECK) expression, an MMP inhibitor, which acts as an endothelial cell suppressor of angiogenesis [33]. TIMP-2 treatment is also able to inhibit endothelial cell growth, by mediating the G1 growth arrest through the activation of the cyclin-dependent kinase inhibitor p27^Kip1^ synthesis, resulting in angiogenesis inhibition [34].

Tumor-bearing TIMP-2 deficient (-/-) mice were found to have a significantly increased number of inflammatory cells in their tumors, as well as increased myeloid-derived suppressor cells (MDSC) coupled with increased MMPs activation and VEGFR1 expression [35]. This same study found that forced TIMP-2 expression in A549 cells significantly reduced recruited MDSCs, which are believed to induce angiogenesis and cancer immunosuppression, in tumors and suppressed angiogenesis and tumor growth. Collectively, the above studies have provided extended mechanistic insights on how TIMP-2 regulates endothelial proliferation and angiogenesis within the TME.

#### Effects on the TME mediated through changes on tumor cell function

For over 20 years now, numerous studies have shown that ectopic expression of TIMP-2 in tumor xenografts or syngeneic models inhibits tumor growth and angiogenesis [27, 40, 41]. There is also growing evidence from clinical-pathological analyses to support that loss of TIMP-2 expression occurs in many types of human cancer, in particular at the late stages of tumor progression [42–44]. However, there are contradicting findings from clinical studies addressing the association of TIMP-2 expression in the development and progression of several cancers [45, 46]. TIMP-2 not only inhibits but also activates MMP-2. Therefore, changes in TIMP-2 and MMP-2 levels (or MMP/TIMP-2 ratio) and activities would also determine whether TIMP-2 role is tumor promoting or inhibitory in a particular type of cancer [47, 48]. More clinical research and mechanistic studies are needed to address this paradox.

It is, however, well documented how TIMP-2 inhibits tumor growth and progression. In a similar mechanism to endothelial cells, TIMP-2 was shown to prevent the activation tyrosine kinase-type receptors (TKR) on human A549 (lung carcinoma), MCF7 (mammary adenocarcinoma), HT1080 fibrosarcoma and Hs68 dermal fibroblast cells [49]. MMPs cleave TGF-α (Transforming Growth Factor alpha), a membrane bound protein that serves as an activating ligand for EGFR (Epidermal Growth Factor Receptor). EGFR is overexpressed in many human cancers and its activation leads to increased cell growth and proliferation through numerous signaling pathways [36]. It was shown that TIMP-2 binding to the cell surface results in increased intracellular cAMP levels. This prevents SHP-1 from dissociating from the EGFR, leading to reduced EGFR phosphorylation, resulting in decreased EGFR activation and growth suppression.

The Focal Adhesion Kinase (FAK) is a signaling protein involved in cellular adhesion and is associated with cellular migration [50]. Elevated FAK phosphorylation correlates with tumor invasiveness and ability to metastasize, where increased FAK activation usually leads to increased cellular migration and invasion. Furthermore, AKT is a key protein kinase involved in important signaling pathways leading to increased cellular proliferation and survival in many cancers [51]. However, as we have previously shown, both FAK and AKT activation (phosphorylation) is inhibited by TIMP-2 through mechanisms other than MMP-2 inhibition [37].

In a recent study, we performed gene expression microarray analysis in lung cancer A549 cells overexpressing TIMP-2 or the mutant Ala + TIMP-2 (devoid of MMP inhibitory activity), to address TIMP-2 mediated transcriptional regulation [38]. The use of Ingenuity Pathway Analysis (IPA), a functional analysis program, enabled us to identify novel biological mechanisms and functions associated with TIMP-2 overexpression in A549 cells. EGF-containing fibulin-like extracellular matrix protein 1 (EFEMP1) is a secreted protein that was determined to inhibit cancerous glioma growth through the reduction of EGFR levels and intracellular AKT phosphorylation [52]. In our system, *EFEMP1* expression was up-regulated in TIMP-2 overexpressing cells, rendering a 6.507 decrease fold-change in cancer development and metastasis-associated functions [38]. In the same study, fibronectin-1, another secreted ECM protein involved in tumor cell growth promotion, was also down regulated upon TIMP-2 forced expression. Finally, forced TIMP-2 expression in A549 cells led to increased *SEMA3A* (Semaphorin-3A) and decreased *ANGPT1* (Angiopoietin 1) transcriptional levels, acting to inhibit angiogenesis pathways [38]. These data suggest that TIMP-2 may regulate the TME by affecting transcription, expression and, therefore, protein secretion important in tumor development and progression.

E-cadherin, a protein involved in cell-cell communication and adhesion, is expressed in all epithelial tissues. However, in many solid malignancies, the expression of E-cadherin is lost, which leads to tumor invasiveness and increased metastatic potential [53]. Furthermore, E-cadherin loss often leads to an epithelial to mesenchymal transition (EMT) phenotype [53]. We have previously demonstrated that TIMP-2 overexpression in lung A549 tumor cells results in up-regulated E-cadherin transcription and protein levels [38]. Additionally, beta-catenin (β-catenin), a protein that directly binds to the C-terminus of the E-cadherin mediating cellular adhesion, was also found to be up-regulated with overexpression of TIMP-2 [38]. EGF has been shown to induce a cell morphology change, EMT, in A549 cells, by internalizing E-cadherin, and down regulating its transcription [54]. However, with TIMP-2 overexpression, both E-cadherin and β-catenin remained strongly localized to the plasma membrane after cell treatment with EGF. These same observations were confirmed upon exogenous treatment with recombinant Ala + TIMP-2 mutant [38]. Taken together, these studies suggest that TIMP-2 promotes an anti-tumor transcriptional profile in cancer cells.

Additionally, the A549 TIMP-2 transcriptome analysis revealed another novel TIMP-2 function [38]. We uncovered that both TIMP-2 and the mutant Ala + TIMP-2 affected the A549 side population (SP) phenotype and functional properties. More specifically, TIMP-2 was determined to decrease the SP in A549 lung cancer cells through decreased transcription of ABCB1 (ATP-binding cassette sub-family G member 1), ABCG2 (ATP-binding cassette sub-family G member 2), and AKR1C1 (Aldo-keto reductase family 1 member C1) [39]. As a result, TIMP-2 overexpression increased A549 tumor cell chemosensitivity to cytotoxic drugs (including doxorubicin and topotecan), suggesting the importance of utilizing TIMP-2 as an inhibitor of tumor growth in combination with well-known chemotherapeutics.

## Conclusions

TIMP-2 provides a novel therapy for cancer treatment. A further understanding of the underlying molecular mechanisms and signaling pathways involving TIMP-2 and the regulation of tumor growth and progression will allow for its full exploitation as a candidate for cancer therapy. As more and more research is being conducted on TIMP-2 molecular mechanisms, its growth inhibitory effects on the tumor cells, and regulation of the tumor microenvironment, this endogenous antitumor and angiogenesis inhibitor may prove to be a powerful new remedy in the ongoing battle against cancer.

## Abbreviations

TME: Tumor microenvironment
ECM: Extracellular matrix
BM: Basement membrane
IGF: Insulin-like growth factor
VEGF: Vascular endothelial growth factor
TGF-β: Transforming growth factor-β
MMP: Matrix metalloproteinase
ADAM: A disintegrin and metalloproteinases
ADAMT: ADAM proteases with thrombospondin motifs
TIMP: Tissue inhibitor of metalloproteinase
VEGFR2: VEGF receptor 2
VEGF-A: Vascular endothelial growth factor A
FGF-2: Fibroblast growth factor 2
α_3_β_1_: Alpha 3 beta 1 integrin receptor
α_v_β_3_: Alpha v beta 3 integrin receptor
PTP: Protein tyrosine phosphatase
IGF-R1: Insulin-like growth factor receptor 1
FGFR1: Fibroblast growth factor receptor 1
VEGFR2: Vascular endothelial growth factor receptor 2
PKA: Protein Kinase A
VE-cadherin: Vascular Endothelial cadherin
RECK: Reversion-inducing-cysteine-rich protein with Kazal motif
MDSC: Myeloid-derived suppressor cells
TKR: Tyrosine kinase-type receptors
TGF-α: Transforming growth factor alpha
EGFR: Epidermal growth factor receptor
FAK: Focal adhesion kinase
IPA: Ingenuity pathway analysis
EFEMP1: EGF-containing fibulin-like extracellular matrix protein 1
SEMA3A: Semaphorin-3A
ANGPT1: Angiopoietin 1
EMT: Epithelial to mesenchymal transition
SP: Side Population
ABCB1: ATP-binding cassette sub-family G member 1
ABCG2: ATP-binding cassette sub-family G member 2
AKR1C1: Aldo-keto reductase family 1 member C1
hMVECs: Human microvascular endothelial cells.

## References

[1] Hanahan D, Weinberg RA (2000). The hallmarks of cancer. Cell.

[2] Egeblad M, Werb Z (2002). New functions for the matrix metalloproteinases in cancer progression. Nat Rev Cancer.

[3] Lu P, Weaver VM, Werb Z (2012). The extracellular matrix: a dynamic niche in cancer progression. J Cell Biol.

[4] Manes S, Mira E, Barbacid MM, Cipres A, Fernandez-Resa P, Buesa JM, Merida I, Aracil M, Marquez G, Martinez AC (1997). Identification of insulin-like growth factor-binding protein-1 as a potential physiological substrate for human stromelysin-3. J Biol Chem.

[5] Bergers G, Brekken R, McMahon G, Vu TH, Itoh T, Tamaki K, Tanzawa K, Thorpe P, Itohara S, Werb Z, Hanahan D (2000). Matrix metalloproteinase-9 triggers the angiogenic switch during carcinogenesis. Nat Cell Biol.

[6] Annes JP, Munger JS, Rifkin DB (2003). Making sense of latent TGFbeta activation. J Cell Sci.

[7] Mott JD, Werb Z (2004). Regulation of matrix biology by matrix metalloproteinases. Curr Opin Cell Biol.

[8] Brinckerhoff CE, Matrisian LM (2002). Matrix metalloproteinases: a tail of a frog that became a prince. Nat Rev Mol Cell Biol.

[9] Sternlicht MD, Werb Z (2001). How matrix metalloproteinases regulate cell behavior. Annu Rev Cell Dev Biol.

[10] Nagase H, Woessner JF (1999). Matrix metalloproteinases. J Biol Chem.

[11] Roy R, Yang J, Moses MA (2009). Matrix metalloproteinases as novel biomarkers and potential therapeutic targets in human cancer. J Clin Oncol.

[12] Coussens LM, Fingleton B, Matrisian LM (2002). Matrix metalloproteinase inhibitors and cancer: trials and tribulations. Science.

[13] Lopez-Otin C, Matrisian LM (2007). Emerging roles of proteases in tumour suppression. Nat Rev Cancer.

[14] Brew K, Nagase H (2010). The tissue inhibitors of metalloproteinases (TIMPs): an ancient family with structural and functional diversity. Biochim Biophys Acta.

[15] Pohar N, Godenschwege TA, Buchner E (1999). Invertebrate tissue inhibitor of metalloproteinase: structure and nested gene organization within the synapsin locus is conserved from Drosophila to human. Genomics.

[16] Jaworski DM, Beem-Miller M, Lluri G, Barrantes-Reynolds R (2007). Potential regulatory relationship between the nested gene DDC8 and its host gene tissue inhibitor of metalloproteinase-2. Physiol Genomics.

[17] Tuuttila A, Morgunova E, Bergmann U, Lindqvist Y, Maskos K, Fernandez-Catalan C, Bode W, Tryggvason K, Schneider G (1998). Three-dimensional structure of human tissue inhibitor of metalloproteinases-2 at 2.1 A resolution. J Mol Biol.

[18] Murphy G (2011). Tissue inhibitors of metalloproteinases. Genome Biol.

[19] Cruz-Munoz W, Khokha R (2008). The role of tissue inhibitors of metalloproteinases in tumorigenesis and metastasis. Crit Rev Clin Lab Sci.

[20] Stetler-Stevenson WG (2008). Tissue inhibitors of metalloproteinases in cell signaling: metalloproteinase-independent biological activities. Science Signal.

[21] Gasson JC, Golde DW, Kaufman SE, Westbrook CA, Hewick RM, Kaufman RJ, Wong GG, Temple PA, Leary AC, Brown EL, Orr EC, Clark SC (1985). Molecular characterization and expression of the gene encoding human erythroid-potentiating activity. Nature.

[22] Maskos K (2005). Crystal structures of MMPs in complex with physiological and pharmacological inhibitors. Biochimie.

[23] Strongin AY, Collier I, Bannikov G, Marmer BL, Grant GA, Goldberg GI (1995). Mechanism of cell surface activation of 72-kDa type IV collagenase. Isolation of the activated form of the membrane metalloprotease. J Biol Chem.

[24] Deryugina EI, Ratnikov B, Monosov E, Postnova TI, DiScipio R, Smith JW, Strongin AY (2001). MT1-MMP initiates activation of pro-MMP-2 and integrin alphavbeta3 promotes maturation of MMP-2 in breast carcinoma cells. Exp Cell Res.

[25] Bourboulia D, Stetler-Stevenson WG (2010). Matrix metalloproteinases (MMPs) and tissue inhibitors of metalloproteinases (TIMPs): Positive and negative regulators in tumor cell adhesion. Semin Cancer Biol.

[26] Stetler-Stevenson WG (2008). The tumor microenvironment: regulation by MMP-independent effects of tissue inhibitor of metalloproteinases-2. Cancer Metastasis Rev.

[27] Seo DW, Li H, Guedez L, Wingfield PT, Diaz T, Salloum R, Wei BY, Stetler-Stevenson WG (2003). TIMP-2 mediated inhibition of angiogenesis: an MMP-independent mechanism. Cell.

[28] Seo DW, Saxinger WC, Guedez L, Cantelmo AR, Albini A, Stetler-Stevenson WG (2011). An integrin-binding N-terminal peptide region of TIMP-2 retains potent angio-inhibitory and anti-tumorigenic activity in vivo. Peptides.

[29] Kim HJ, Cho YR, Kim SH, Seo DW (2014). TIMP-2-derived 18-mer peptide inhibits endothelial cell proliferation and migration through cAMP/PKA-dependent mechanism. Cancer Lett.

[30] Barazi HO, Zhou L, Templeton NS, Krutzsch HC, Roberts DD (2002). Identification of heat shock protein 60 as a molecular mediator of alpha 3 beta 1 integrin activation. Cancer Res.

[31] Kim SH, Cho YR, Kim HJ, Oh JS, Ahn EK, Ko HJ, Hwang BJ, Lee SJ, Cho Y, Kim YK, Stetler-Stevenson WG, Seo DW (2012). Antagonism of VEGF-A-induced increase in vascular permeability by an integrin alpha3beta1-Shp-1-cAMP/PKA pathway. Blood.

[32] Fernandez CA, Roy R, Lee S, Yang J, Panigrahy D, Van Vliet KJ, Moses MA (2010). The anti-angiogenic peptide, loop 6, binds insulin-like growth factor-1 receptor. J Biol Chem.

[33] Oh J, Seo DW, Diaz T, Wei B, Ward Y, Ray JM, Morioka Y, Shi S, Kitayama H, Takahashi C, Noda M, Stetler-Stevenson WG (2004). Tissue inhibitors of metalloproteinase 2 inhibits endothelial cell migration through increased expression of RECK. Cancer Res.

[34] Seo DW, Li H, Qu CK, Oh J, Kim YS, Diaz T, Wei B, Han JW, Stetler-Stevenson WG (2006). Shp-1 mediates the antiproliferative activity of tissue inhibitor of metalloproteinase-2 in human microvascular endothelial cells. J Biol Chem.

[35] Guedez L, Jensen-Taubman S, Bourboulia D, Kwityn CJ, Wei B, Caterina J, Stetler-Stevenson WG (2012). TIMP-2 targets tumor-associated myeloid suppressor cells with effects in cancer immune dysfunction and angiogenesis. J Immunother.

[36] Fischer OM, Hart S, Gschwind A, Ullrich A (2003). EGFR signal transactivation in cancer cells. Biochem Soc Trans.

[37] Bourboulia D, Jensen-Taubman S, Rittler MR, Han HY, Chatterjee T, Wei B, Stetler-Stevenson WG (2011). Endogenous angiogenesis inhibitor blocks tumor growth via direct and indirect effects on tumor microenvironment. Am J Pathol.

[38] Bourboulia D, Han H, Jensen-Taubman S, Gavil N, Isaac B, Wei B, Neckers L, Stetler-Stevenson WG (2013). TIMP-2 modulates cancer cell transcriptional profile and enhances E-cadherin/beta-catenin complex expression in A549 lung cancer cells. Oncotarget.

[39] Han H, Bourboulia D, Jensen-Taubman S, Isaac B, Wei B, Stetler-Stevenson WG (2014). An endogenous inhibitor of angiogenesis inversely correlates with side population phenotype and function in human lung cancer cells. Oncogene.

[40] Hajitou A, Sounni NE, Devy L, Grignet-Debrus C, Lewalle JM, Li H, Deroanne CF, Lu H, Colige A, Nusgens BV, Frankenne F, Maron A, Yeh P, Perricaudet M, Chang Y, Soria C, Calberg-Bacq CM, Foidart JM, Noël A (2001). Down-regulation of vascular endothelial growth factor by tissue inhibitor of metalloproteinase-2: effect on in vivo mammary tumor growth and angiogenesis. Cancer Res.

[41] Imren S, Kohn DB, Shimada H, Blavier L, DeClerck YA (1996). Overexpression of tissue inhibitor of metalloproteinases-2 retroviral-mediated gene transfer in vivo inhibits tumor growth and invasion. Cancer Res.

[42] Ara T, Fukuzawa M, Kusafuka T, Komoto Y, Oue T, Inoue M, Okada A (1998). Immunohistochemical expression of MMP-2, MMP-9, and TIMP-2 in neuroblastoma: association with tumor progression and clinical outcome. J Pediatr Surg.

[43] Wajner SM, Capp C, Brasil BA, Meurer L, Maia AL (2014). Reduced tissue inhibitor of metalloproteinase-2 expression is associated with advanced medullary thyroid carcinoma. Oncol Lett.

[44] Alakus H, Grass G, Hennecken JK, Bollschweiler E, Schulte C, Drebber U, Baldus SE, Metzger R, Holscher AH, Monig SP (2008). Clinicopathological significance of MMP-2 and its specific inhibitor TIMP-2 in gastric cancer. Histol Histopathol.

[45] Remacle A, McCarthy K, Noel A, Maguire T, McDermott E, O’Higgins N, Foidart JM, Duffy MJ (2000). High levels of TIMP-2 correlate with adverse prognosis in breast cancer. Int J Cancer.

[46] Kallakury BV, Karikehalli S, Haholu A, Sheehan CE, Azumi N, Ross JS (2001). Increased expression of matrix metalloproteinases 2 and 9 and tissue inhibitors of metalloproteinases 1 and 2 correlate with poor prognostic variables in renal cell carcinoma. Clin Cancer Res.

[47] Branca M, Ciotti M, Giorgi C, Santini D, Di Bonito L, Costa S, Benedetto A, Bonifacio D, Di Bonito P, Paba P, Accardi L, Syrjänen S, Favalli C, Syrjänen K (2006). HPV-PathogenISS Study Group: Matrix metalloproteinase-2 (MMP-2) and its tissue inhibitor (TIMP-2) are prognostic factors in cervical cancer, related to invasive disease but not to high-risk human papillomavirus (HPV) or virus persistence after treatment of CIN. Anticancer Res.

[48] Fan YZ, Zhang JT, Yang HC, Yang YQ (2002). Expression of MMP-2, TIMP-2 protein and the ratio of MMP-2/TIMP-2 in gallbladder carcinoma and their significance. World J Gastroenterol.

[49] Hoegy SE, Oh HR, Corcoran ML, Stetler-Stevenson WG (2001). Tissue inhibitor of metalloproteinases-2 (TIMP-2) suppresses TKR-growth factor signaling independent of metalloproteinase inhibition. J Biol Chem.

[50] Zhao J, Guan JL (2009). Signal transduction by focal adhesion kinase in cancer. Cancer Metastasis Rev.

[51] Fresno Vara JA, Casado E, de Castro J, Cejas P, Belda-Iniesta C, Gonzalez-Baron M (2004). PI3K/Akt signalling pathway and cancer. Cancer Treat Rev.

[52] Hu Y, Pioli PD, Siegel E, Zhang Q, Nelson J, Chaturbedi A, Mathews MS, Ro DI, Alkafeef S, Hsu N, Hamamura M, Yu L, Hess KR, Tromberg BJ, Linskey ME, Zhou YH (2011). EFEMP1 suppresses malignant glioma growth and exerts its action within the tumor extracellular compartment. Mol Cancer.

[53] Ceteci F, Ceteci S, Karreman C, Kramer BW, Asan E, Gotz R, Rapp UR (2007). Disruption of tumor cell adhesion promotes angiogenic switch and progression to micrometastasis in RAF-driven murine lung cancer. Cancer Cell.

[54] Lu Z, Ghosh S, Wang Z, Hunter T (2003). Downregulation of caveolin-1 function by EGF leads to the loss of E-cadherin, increased transcriptional activity of beta-catenin, and enhanced tumor cell invasion. Cancer Cell.

